# Fecal virome analysis of three carnivores reveals a novel nodavirus and multiple gemycircularviruses

**DOI:** 10.1186/s12985-015-0305-5

**Published:** 2015-05-20

**Authors:** Nádia Conceição-Neto, Mark Zeller, Elisabeth Heylen, Hanne Lefrère, João Rodrigo Mesquita, Jelle Matthijnssens

**Affiliations:** Laboratory of viral metagenomics, Rega Institute for Medical Research Leuven, Department of Microbiology and Immunology, KU Leuven - University of Leuven, Leuven, B-3000 Belgium; Polytechnic Institute of Viseu, Department of Animal Science, Rural Engineering and Veterinary, Viseu, Portugal

**Keywords:** Virome, Gemycircularvirus, Metagenomics, viral discovery

## Abstract

**Background:**

More knowledge about viral populations in wild animals is needed in order to better understand and assess the risk of zoonotic diseases. In this study we performed viral metagenomic analysis of fecal samples from three healthy carnivores: a badger (*Meles meles*), a mongoose (*Herpestes ichneumon*) and an otter (*Lutra lutra*) from Portugal.

**Results:**

We detected the presence of novel highly divergent viruses in the fecal material of the carnivores analyzed, such as five gemycircularviruses. Four of these gemycircularviruses were found in the mongoose and one in the badger. In addition we also identified an RNA-dependent RNA polymerase gene from a putative novel member of the *Nodaviridae* family in the fecal material of the otter.

**Conclusions:**

Together these results underline that many novel viruses are yet to be discovered and that fecal associated viruses are not always related to disease. Our study expands the knowledge of viral species present in the gut, although the interpretation of the true host species of such novel viruses needs to be reviewed with great caution.

## Background

With the advent of next generation sequencing techniques, samples from a wide range of animal species have been screened to identify novel viruses and this approach has become the most important tool for early detection and characterization of possible emerging zoonotic agents [1–3]. It is important to monitor these emerging zoonotic agents as they can be responsible for minor or major epidemics worldwide [4]. For example, zoonoses can range from the Middle East respiratory syndrome coronavirus (MERS-CoV), which recently drew a lot of attention worldwide [5], to the 2009 pandemic H1N1 influenza A virus or to the less publicized Hepatitis E virus [6]. Not only are humans at risk, but also animals can be infected with viruses from other host species, resulting in disease, or further transmission to humans. The severe acute respiratory syndrome (SARS) coronavirus pandemic originated from wildlife, where bats where identified as the reservoir and civets as an intermediate host [7, 8]. A similar example comes from Hendra virus, which also originated from bats, but transmission to humans occurred with horses as an intermediate host, causing severe pneumonia in horses and humans [9]. As such, zoonotic infections can have significant consequences for animal and public welfare. However, little is known about these pathogens before they emerge from unrecognized zoonotic sources and therefore a deeper understanding of the virome of wild animals will allow us to more rapidly identify the host of particular novel zoonotic viruses, and act appropriately to prevent further spread of such viruses.

Fourteen species of wild carnivores can be found in Portugal, often in relative close contact with humans. Only red fox, stone marten, badger, common genet and, more recently, the Egyptian mongoose, are known to have a generalized distribution throughout the country [10]. From these animals we sampled two species, a badger (*Meles meles*) and an Egyptian mongoose (*Herpestes ichneumon*). In addition, we sampled a Eurasian otter (*Lutra lutra*) raised in a zoo, since otters are also widely distributed in Portugal [11]. Currently little is known about the viral communities that populate the gut of these animals. Thus far, Bodewes and colleagues investigated the fecal virome of badgers and an otter from Spain, and found a fecal phlebovirus in an otter [12]. A study by van den Brand and colleagues investigated the virome of badgers in the Netherlands, identifying two novel circularviruses [13]. In Portugal, a study performed by Oliveira and colleagues screened otters for parvoviruses, adenoviruses and parainfluenza virus, however none of the samples had detectable levels of virus [11]. Also in Portugal, another study screened for and identified parvoviruses in genets, badgers and mongooses [10]. As these findings are probably only the tip of the iceberg, we were interested to further explore the viral communities of the gut in widely spread wild species in Portugal and a zoo specimen.

## Results and discussion

### Identification of five novel gemycircularviruses in the fecal samples from the badger and mongoose

In recent years, ssDNA viruses have been frequently found in fecal samples of a variety of animal species, such as badgers [13], bats [14], cows [15], turkey [16], rodents [17, 18], chimpanzees [19], pigs [20–22], New Zealand fur seal [20] foxes [23] and recently also in ancient caribou feces [24]. ssDNA viruses are small viruses (1.4-8.5 kb) and can encode as little as two proteins: a capsid and a replication associated protein. Thus far, seven major ssDNA families have been identified based on the host range and type of ssDNA genome. Only the *Parvoviridae* family encodes for a linear genome, the remaining are circular. The *Nanoviridae* and *Geminiviridae* families infect plants, whereas *Circoviridae*, *Parvoviridae* and *Anelloviridae* are known to infect animals. Viruses belonging to the *Inoviridae* and *Microviridae* infect bacteria [25].

**Fig. 1:**
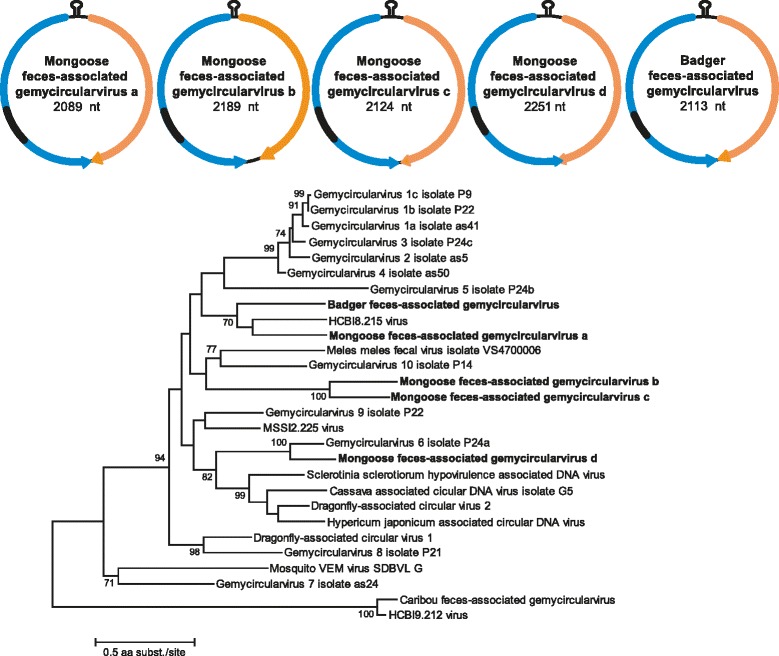
Genome organization of novel gemycircularviruses and maximum likelihood phylogenetic tree of the REP of gemycircularviruses. Depicted in blue is the Rep with an intron in black and depicted in orange is the capsid protein. Bootstrap values even or greater than 70 are shown

In our study, four complete novel gemycircularviruses were found in the mongoose and one in the badger feces. The first gemycircularvirus was discovered in 2010 in fungi [26]. Since then, these novel ssDNA circular viruses have been found in a wide range of hosts, including the cassava plant [27], badger feces [13], mosquitoes [25], dragonflies [28], *Hypericum japonicum*, a flowering plant in the family Hypericaceae [29], as well as in the gut of a wide variety of mammals and birds from New Zealand [30]. Although these viruses were initially named gemini-like viruses, the novel genus gemycircularvirus was recently proposed by Rosario and colleagues [28]. Gemycircularviruses encode for a highly variable capsid protein and a more conserved replication associated protein (Rep). Figure 1 shows the phylogenetic analysis of known gemycircularviruses based on the amino acid level of the Rep gene. The viruses identified in this study showed to be distantly related to the currently known gemycircularviruses. The first gemycircularvirus found in the mongoose, named ‘Mongoose feces-associated gemycircularvirus a’, was found to be most closely related to the Badger feces-associated gemycircularvirus, sharing 57.2 % similarity on the amino-acid level. On the amino-acid level, ‘Mongoose feces-associated gemycircularvirus b’ shares its highest similarity (61.0 %) with ‘Mongoose feces-associated gemycircularvirus c’. The most closely related virus to the ‘Mongoose feces-associated gemycircularvirus d’ shares 71.7 % amino-acid similarity, and was found in the New Zealand bird Chatham gerygone (Gemycircularvirus 6 isolate P24a). All five gemycircularviruses contained nonanucleotide stem loop motifs, conserved Rolling Circle Replication (RCR) motifs (I, II, III and geminivirus-like Rep sequence motif (GRS)) and helicase motifs (Walker-A and Walker-B) (Table 1). Only the Mongoose feces-associated gemycircularvirus d had a nonanucleotide motif identical to the one found in most known gemycircularviruses (TAATATTAT), but the Rep motifs of the newly discovered viruses also showed a variable degree of similarity among each other (Table 1) and with previously characterized gemycircularviruses [28, 20]. The discovery of these novel viruses expands the knowledge on gemycircularviruses genetic diversity and their putative host range. Initially, it was hypothesized that these viruses infect fungi, since one of these viruses is known to infect the pathogenic fungi *Sclerotinia sclerotiorum* [30], however it is not known whether the remaining viruses do. Most recently, clones from a novel gemycircularvirus found in caribou feces were inoculated in a plant, resulting in a successful infection [24]. Nevertheless, the true host(s) of these viruses remain to be determined. In our case, these viruses might have infected the mongoose and the badger, or alternatively, might have arisen from fungi inhabiting their intestines, or they could also be derived from insects or plants as part of the diet of the badger and the mongoose. Therefore, since the true host cannot be determined yet, the nomenclature of these novel viruses should be addressed cautiously. We tentatively named them feces-associated gemycircularviruses, preceded by the common name of the animal where they were isolated from.

**Table 1:** Motifs of the Rep from the novel gemycircularviruses and reference gemycircularviruses

Virus name	Size (nt)	Nonanucleotide motif	Motif I	Motif II	GRS	Motif III	Walker-A	Walker-B
Mongoose feces-associated gemycircularvirus a	2089	TATAAATAC	LLTYA	HLHSFID	DIFDVDGCHPNVSPTH	YACKD	GPSRMGKT	VFDDI
Mongoose feces-associated gemycircularvirus b	2189	TATAAATAC	LFTYS	HYHVFDV	RKFDVEGFHPNIVPSL	YATKD	GRSKTGKT	VFDDI
Mongoose feces-associated gemycircularvirus c	2124	TAATATTAC	LFTYS	HLHAFVD	RKFDVEGFHPNIISTS	YATKD	GPSRTGKT	VFDDI
Mongoose feces-associated gemycircularvirus d	2251	TAATATTAT	LLTYA	HLHCFVD	RVFDVGGFHPNISPSR	YAIKD	GRSLTGKT	VLDDV
Badger feces-associated gemycircularvirus	2113	TAATACTAT	LLTYA	HLHAFVH	TVFDVAGFHPNISPSF	YAIKD	GPSRVGKT	VFDDI
MSSI2.225 virus	2259	TAATGTTAT	LLTYP	HLHAFVD	RAFDVEGCHPNVSPSP	YAIKD	GGKLSCTS	IFDDF
Fecal associated gemycircularvirus 1a	2197	TAATATTAT	LLTYA	HLHAFVD	DVFDVGGRHPNLVPSY	YAIKD	GDTRLGKT	VFDDM
Faecal associated gemycircularvirus 4	2224	TAATGTTAT	LLTYA	HLHAFCD	DVFDVGGFHPNIEASR	YAIKD	GDTRLGKT	VFDDM
Fecal associated gemycircularvirus 5	2187	TAATATTAT	LVTYP	HLHVFCD	DIFDVGGFHPNIERSK	YACKD	GDALTGKT	VIDDI
Cassava-associated circular DNA virus	2220	TAATATTAT	LITYA	HLHCFID	DIFDVDGRHPNIEPSW	YAIKD	GDSRSGKT	IFDDI
Dragonfly-associated circular DNA virus-2	2236	TAATATTAT	LVTYP	HLHCFAD	DIFDVDGCHPNIQPST	YAIKD	GESRTGKT	IFDDI
Mosquito VEM virus SDBVL G	2238	TAATATTAT	LLTYA	HFHAFLD	RFWDIAGRHPNIARVG	YAIKD	GPSRTGK?	VFDDI

### Identification a partial RNA-dependent RNA polymerase of a nodavirus in the fecal material of the otter

**Fig. 2:**
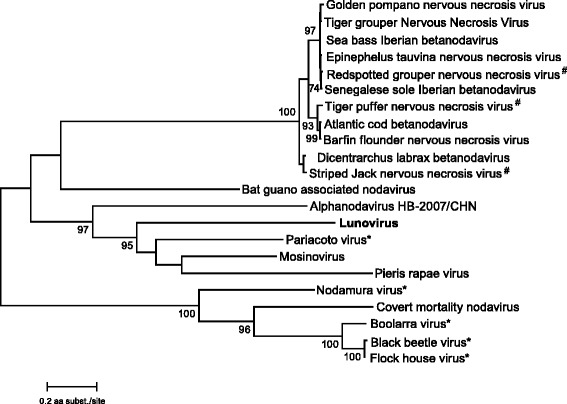
Maximum-likelihood phylogenetic tree of the RNA1 of several *Nodaviridae* and Lunovirus. The tree represents viral members of the *Nodaviridae* and the partial RNA1 of the Lunovirus identified in the otter (Bootstrap values even or greater than 70 are shown). Viruses represented with an asterisk (*) have been currently recognized as Alphanodaviruses and viruses represented with a number sign (*#*) have been recognized as Betanodaviruses by the ICTV [34]

The *Nodaviridae* family comprises two genera *Alphanodavirus* and the *Betanodavirus* of bipartite single stranded RNA viruses [31]. Alphanodaviruses are usually insect viruses whereas betanodaviruses infect fish and are responsible for viral nervous necrosis in numerous fish species [32, 33]. Nodaviruses have two segments, RNA1 (3.2 kb) encodes for a RNA-dependent RNA polymerase (RdRp) responsible for its RNA replication and RNA2 (1.2 kb) encodes for a capsid protein [31]. Nodaviruses are classified by the ICTV (International Committee on Taxonomy of Viruses) according to the genetic diversity of the RNA2 segment [34]. In the fecal material of the otter we identified a partial RNA1 of a novel putative Nodavirus (1.7 kb), which was most closely related to the recently discovered Mosinovirus, isolated from mosquitoes, sharing 43 % similarity on the amino-acid level (Fig. 2). Adopting the convention of naming based on Schuster and colleagues for Mosinovirus virus (mosquito nodavirus), we have tentatively named the virus Lunovirus (*Lutra lutra* nodavirus) [35]. As the RNA2 of Lunovirus was not found, most likely due to the fact that it is highly divergent and could not be detected by similarity searches in current viral databases, we should be reluctant to suggest a final classification. However, based on the large divergence of the Lunovirus RNA1 with the RNA1 of other nodaviruses, it seems likely that the Lunovirus is a novel member of the *Nodaviridae* family.

## Conclusions

In the three healthy carnivores analyzed, viral sequences belonging to the *Caudovirales* order of bacteriophages were also detected, as previously reported [12]. Furthermore, our study showed that even healthy wildlife seems to harbor many divergent viral communities that deserve to be explored further to expand our current knowledge and databases. From the limited data available from the fecal virome studies from an otter (*Lutra lutra*) and badger (*Meles meles*) of Bodewes and van der Brand [12, 13], the virus families discovered in our study are completely different, possibly resulting from different diets, or indicating a large unexplored area of the ‘virome space’. Also the fact that the viruses discovered are highly different from viruses available in databases, might explain the difficulty in finding viruses by regular PCR screening, as previously reported [11]. These novel viruses reported in this study are likely derive from the diet, as the Nodavirus from the otter is likely to be from fish and the gemycircularviruses from insects, which are part of the animals diets. Viral discovery can be challenging because novel viruses, as seen in this study, can be quite divergent and their classification and true host determination can be difficult. In this regard, replication associated proteins have shown to be conserved, and the best strategy to create alignments. It is very interesting to see the ubiquity of different circular virus species found nowadays due to the availability of next-generation sequencing. Screening of larger groups of animals and species will help to increase our knowledge of viruses circulating in wild animals.

## Methods

### Sample collection

Fecal samples were collected from a badger (*Meles meles*), a mongoose (*Herpestes ichneumon*) from a rescue center and an Eurasian otter (*Lutra lutra*) from a zoo upon their arrival in 2011 at the wildlife center “Parque Biológico da Serra da Lousã” in Coimbra district, Portugal. This collection was part of the quarantine assessment program applied to newly introduced animals in the center. Samples were kept at −80 °C until further processing.

### Sample preparation

Ten percent fecal suspensions were homogenized for 1 min at 3000 rpm with a MINILYS homogenizer (Bertin Technologies) and filtered consecutively through 100 μm, 10 μm and 0.8 μm membrane filters (Millipore) for 30 s at 1250 *g*. The filtrate was then treated with a homemade buffer (1 M Tris, 100 mM CaCl_2_ and 30 mM MgCl_2_) and a cocktail of Benzonase (Novagen) and Micrococcal Nuclease (New England Biolabs) at 37 °C for 2 h to digest free-floating nucleic acids. RNA and DNA was extracted using the QIAamp Viral RNA Mini Kit (Qiagen) according to the manufacturer’s instructions but without addition of carrier RNA to the lysis buffer. First and second strand synthesis and random PCR amplification for 25 cycles were performed using a slightly modified Whole Transcriptome Amplification (WTA) Kit procedure (Sigma-Aldrich), with a denaturation temperature of 95 °C instead of 72 °C to allow for the denaturation of dsDNA and dsRNA. This modification leads to the amplification of both RNA and DNA. A size selection after library synthesis was performed using a 0.7 ratio of Agencourt AMPure XP beads (Beckman Coulter, Inc.). WTA products were purified with MSB Spin PCRapace spin columns (Stratec) and were prepared for Illumina sequencing using the KAPA Library Preparation Kit (Kapa Biosystems). Fragments ranging from 350–600 bp were selected using the BluePippin (Sage Science) according to the manufacturer’s instructions. Libraries were quantified with the KAPA Library Quantification kit (Kapa Biosystems) and sequencing of the samples was performed on a HiSeq 2500 platform (Illumina) for 301 cycles (150 bp paired ends). Each sample was attributed a total of 2 million paired end reads. Mongoose feces associated gemycircularvirus a, b, c and d yielded 262, 295, 37,644, and 356 reads respectively. The Badger feces associated gemycircularvirus yielded 88 reads and the RNA2 of the Nodavirus 20 reads.

### Genomic and phylogenetic analysis

Raw reads were trimmed for quality and adapters using Skewer [36] and were *de novo* assembled into contigs using SPAdes [37]. Scaffolds were classified using a tBLASTx search against all complete viral genomes in GenBank using an e-value cut-off of 10^−10^. Scaffolds with a significant tBLASTx hit were retained and used for a second tBlastx search against the GenBank nucleotide database using an e-value of 10^−4^ [38]. Open reading frames (ORF) were identified with ORF Finder analysis tools and the conserved motifs in the amino acid sequences were identified with HMMER [39]. Amino acid alignments of the viral sequences were performed with MAFFT version 7 [40] using the --auto option. Maximum likelihood phylogenetic trees were constructed in MEGA6.0 [41], using JCC (best substitution model) with 500 bootstrap replicates. Potential intron acceptor and donor sites from the novel gemycircularviruses were identified manually. Using the method above we were able to retrieve the five complete gemycircularvirus and partial sequence of the RNA2 of the Lunovirus. Presence of the discovered novel viruses was then confirmed by PCR and Sanger sequencing using the original extracts. Gemycircularviruses’ primers were designed in the capsid gene, covering the complete circular genome and the nodavirus primer pair targeted the RNA2 sequence found. All sequences from the novel viruses were submitted to GenBank [KP263543, KP263544, KP263543-KP263548, KP263546, KP263547, KP263548].
